# c-di-AMP accumulation impairs toxin expression of *Bacillus anthracis* by down-regulating potassium importers

**DOI:** 10.1128/spectrum.03786-23

**Published:** 2024-06-20

**Authors:** Jia Hu, Junmin Yao, Chengfeng Lei, Xiulian Sun

**Affiliations:** 1 Wuhan Institute of Virology, Center for Biosafety Mega-Science, Chinese Academy of Sciences, Wuhan, Hubei, China; 2 University of Chinese Academy of Sciences, Beijing, China; South China Sea Institute of Oceanology, Guangzhou, China

**Keywords:** potassium importer, c-di-AMP receptors, anthrax toxin, KtrC, KdpD

## Abstract

The Gram-positive bacterium *Bacillus anthracis* is the causative agent of anthrax and a bioterrorism threat worldwide. As a crucial second messenger in many bacterial species, cyclic di-AMP (c-di-AMP) modulates various key processes for bacterial homeostasis and pathogenesis. Overaccumulation of c-di-AMP alters cellular growth and reduces anthrax toxin expression as well as virulence in *Bacillus anthracis* by unresolved underlying mechanisms. In this report, we discovered that c-di-AMP binds to a series of receptors involved in potassium uptake in *B. anthracis*. By analyzing Kdp and Ktr mutants for osmotic stress, gene expression, and anthrax toxin expression, we also showed that c-di-AMP inhibits Kdp operon expression through binding to the KdpD and *ydaO* riboswitch; up-regulating intracellular potassium promotes anthrax toxin expression in c-di-AMP accumulated *B. anthracis*. Decreased anthrax toxin expression at high c-di-AMP occurs through the inhibition of potassium uptake. Understanding the molecular basis of how potassium uptake affects anthrax toxin has the potential to provide new insight into the control of *B. anthracis.*

**IMPORTANCE:**

The bacterial second messenger cyclic di-AMP (c-di-AMP) is a conserved global regulator of potassium homeostasis. How c-di-AMP regulates bacterial virulence is unknown. With this study, we provide a link between potassium uptake and anthrax toxin expression in *Bacillus anthracis*. c-di-AMP accumulation might inhibit anthrax toxin expression by suppressing potassium uptake.

## INTRODUCTION

Establishing a successful bacterial infection requires several crucial steps, including attachment to the host cell, immune evasion, and release of virulence factors or toxins. The protein enzymes and virulence factors involved in these steps are tightly regulated by different signal transduction pathways. Many transduction pathways are regulated by cyclic nucleotide second messengers (1–8). Among these second messengers, cyclic di-3′,5′-AMP (c-di-AMP) has been implicated in bacterial physiology and pathogenesis because it regulates diverse cellular processes (9–16).

Synthesized by diadenylate cyclase domain (DAC)-carrying proteins, c-di-AMP is bound by a variety of c-di-AMP receptors and is either degraded or exported from the cell by specific phosphodiesterases (2). Several c-di-AMP receptors have been identified. The TetR transcriptional factor DarR in *Mycobacterium smegmatis* (17), PII-like PstA protein in *Staphylococcus aureus* (18), and pyruvate carboxylase LmPC in *Listeria monocytogenes* (19) were reported to control various cellular processes from fatty acid to carbon metabolism. In contrast to these diverse c-di-AMP effectors with non-conserved or unidentified ligand binding signatures, the major group of c-di-AMP receptors, e.g., KtrA, CpaA, KhtT, and BusR, all carry an RCK_C (regulator of conductance of K^+^ C-terminal) or CBS (cystathionine-beta synthase) domain as conserved c-di-AMP binding sites, implicating their direct involvement in potassium or osmolyte transport (2). In *Bacillus* and *Clostridium*, c-di-AMP directly interacts with riboswitch *ydaO* to attenuate the expression of the potassium transport system (20, 21). Although the molecular details through which c-di-AMP regulates K^+^ channel activity or alters channel abundance differs across species, the roles of c-di-AMP in regulating the intracellular potassium level are evolutionarily conserved in *Bacillus subtilis*, *Bacillus thuringiensis*, *S. aureus*, *Lactococcus lactis*, *L. monocytogenes,* and *Streptococcus pneumoniae* (22–27).

As the most abundant bacterial intracellular cation, potassium plays a critical role in bacterial physiology (28). Potassium functions as a compatible solute under hyperosmotic shock, modulates resistance to antimicrobials, and is optimal for the activity of various enzymes, including the ribosome. In contrast, potassium accumulation may be toxic if the intracellular concentration is too high. Therefore, K^+^ homeostasis must be carefully controlled in bacteria. Various bacteria possess passive potassium channels or active transporters to maintain an appropriate concentration of potassium ions (29). K^+^ homeostasis has been implicated in the virulence of several pathogenic bacteria (29). The K^+^ transporters Kdp and Kup are required for *Salmonella enterica* invasion into colonic epithelial cells and important for the secretion of the effector proteins of the Type III secretion system of *Salmonella* pathogenicity island 1 (30). Deletion of the potassium translocating Trk system in *S. enterica* or *Mycobacterium tuberculosis* decreases virulence in mice (31, 32). The absence of the Ktr system in *S. aureus* reduces survival under hyperosmotic conditions and increases cell susceptibility to antibiotics (33).

The Gram-positive model bacterium *B. subtilis* encodes three potassium uptake systems, the high- and low-affinity potassium channels KtrAB and KtrCD, respectively, and the high-affinity K^+^/H^+^ symporter KimA (20, 34). The expression of the KimA and KtrAB operon is repressed by a riboswitch that responds to the second messenger c-di-AMP. c-di-AMP not only interferes with the expression of the K^+^ uptake systems but also inactivates KtrAB and KtrCD by binding directly to the C-lobe of KtrA and KtrC. Mechanisms of K^+^ uptake have been well characterized in the model organism *B. subtilis*. However, no studies have demonstrated the mechanism of K^+^ uptake in the pathogenic *Bacillus anthracis. B. subtilis* relies on well-described high-affinity transporters KtrAB and KimA and the low-affinity transporter KtrCD to maintain the intracellular pool of potassium (34). In contrast, a BLAST P sequence analysis revealed that the *B. anthracis* genome encodes KdpFA/B/C/D (BA_5871 and BA_0739–BA_0742) and KtrCB/CD (BA_4191, BA_1323, and BA_1333). The gene encoding KdpE is absent in the genome of *B. anthracis,* and KdpD is truncated. The Kim A was identified as a high-affinity potassium transporter in *L. monocytogenes* and *S. aureus*, whereas no close protein homolog was detected in the *B. anthracis* genome (20). A riboswitch was identified by BLAST N upstream of KdpFABC. However, the functions of the two potassium uptake systems, particularly how they interwind with global c-di-AMP signaling, have not been studied in *B. anthracis*.

*B. anthracis* is a spore-forming microbe that causes the zoonotic disease anthrax. The anthrax toxins and a capsule are two major virulence factors responsible for bacterial virulence (35). The three subunits of the tripartite anthrax toxin are protective antigen (PA), lethal factor (LF), and edema factor (EF). Non-toxic LF and EF in combination with PA form two toxins producing diverse pathogenic responses (36). c-di-AMP signaling has been previously studied in *B. anthracis* (37). In *B. anthracis*, c-di-AMP is synthesized by three enzymes (DisA, CdaA, and CdaS) and degraded by two phosphodiesterases GdpP and PgpH. The inactivation of GdpP and PgpH increased intracellular c-di-AMP concentration, decreased bacterial toxin expression, and attenuated virulence in the mouse model.

In this report, we identified KtrC and KdpD, two potassium uptake proteins, as direct targets of the signaling nucleotide c-di-AMP in *B. anthracis*. Our study showed that c-di-AMP accumulation indirectly represses anthrax toxin expression by inhibiting potassium uptake. We further identified the function of KtrC and KdpD. KtrC is required for osmotic stress resistance, whereas both deletion and overexpression of *kdpD* increased *ktr* operon expression.

## RESULTS

### Identification of c-di-AMP receptor proteins

Recently, several receptors that bind c-di-AMP have been identified. Most of these receptors regulate potassium or compatible solute levels. Because our previous results showed that c-di-AMP accumulation impairs anthrax toxin expression (37), we hypothesized that c-di-AMP-bound potassium transporters regulate anthrax toxin expression. Consequently, the *B. anthracis* Sterne genome was analyzed for genes encoding putative potassium transporters and proteins related to osmotic regulation. A potential c-di-AMP receptor KdpD, five proteins carrying RCK_C domains, and eight containing CBS domains were selected as potential c-di-AMP binding protein candidates in *B. anthracis*.

The lysates of the strains carrying the corresponding plasmids were analyzed for a possible interaction with ^32^P-labeled c-di-AMP *in vitro* using the differential radial capillary action of ligand assay (DraCALA) (38).

**Fig 1:**
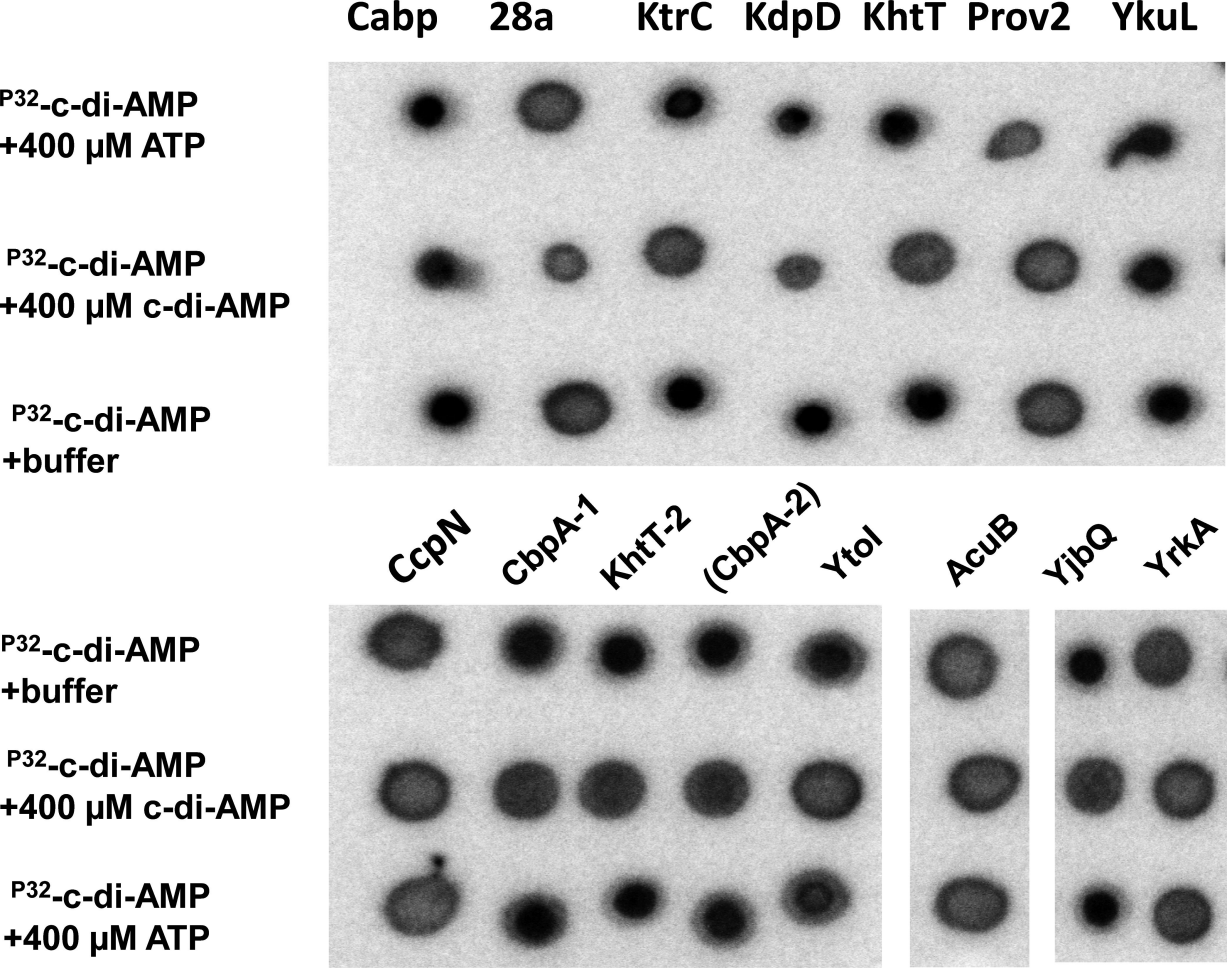
c-di-AMP-interacting proteins determined by DraCALA. DraCALAs with ^32^P-labeled c-di-AMP and *Escherichia coli* extracts prepared from the vector control strain (pET28a) or strains overproducing potential c-di-AMP receptors. Cold c-di-AMP/ATP was added as a competitor where indicated. KtrC (BA_4191), KhtT (BA_5287), KhtT-2 (BA_4994), and YjbQ (BA_0699) are RCK domain-containing proteins. Prov2 (BA_2786), YkuL (BA_4196), YrkA (BA_3422), AcuB (BA_4917), CcpN (BA_4521), YtoI (BA_4858), CbpA-1 (BA_2601), and CbpA-2 (BA_1348) are CBS domain-containing proteins.

In the initial screen, nine target proteins were identified because they enriched c-di-AMP ligands in the DraCALA (Fig. 1). Among these proteins, the known c-di-AMP binding protein CabP served as the positive control (25). Four proteins (KtrC, KhtT-1, KhtT-2, and YjbQ) containing an RCK_C domain were identified as c-di-AMP binding proteins. KtrC, the homolog of KtrC in *B. subtilis*, and the cytoplasmic subunit KhtT/KhtT2 and YjbQ, a putative potassium transporter, showed binding to c-di-AMP. Four proteins (YkuL, YtoI, CbpA-1, and CbpA-2) with the CBS domain were identified as c-di-AMP binding proteins. YkuL is a homolog of CbpB (DarB) in *B. subtilis*, which controls (p)ppGpp synthesis (39). YtoI and two CbpA proteins are c-di-AMP receptors with unknown functions. CbpA, a homolog of CbpA in *L. monocytogenes* (19), showed strong binding to c-di-AMP. KdpD is a homolog of KdpD in *S. aureus* with strong binding to c-di-AMP. ATP did not block c-di-AMP binding for all proteins tested, except YkuL, whereas 400 µM cold c-di-AMP outcompeted the radioactively labeled c-di-AMP (Fig. 1). This result demonstrates the specificity of c-di-AMP binding.

In total, we identified nine c-di-AMP receptor proteins in *B. anthracis*. Five of these proteins (KtrC, YjbQ, KhtT-1, KhtT-2, and KdpD) are involved in potassium uptake and export.

### c-di-AMP inhibits KtrCB/D and KdpFABC potassium transport activity

**Fig 2:**
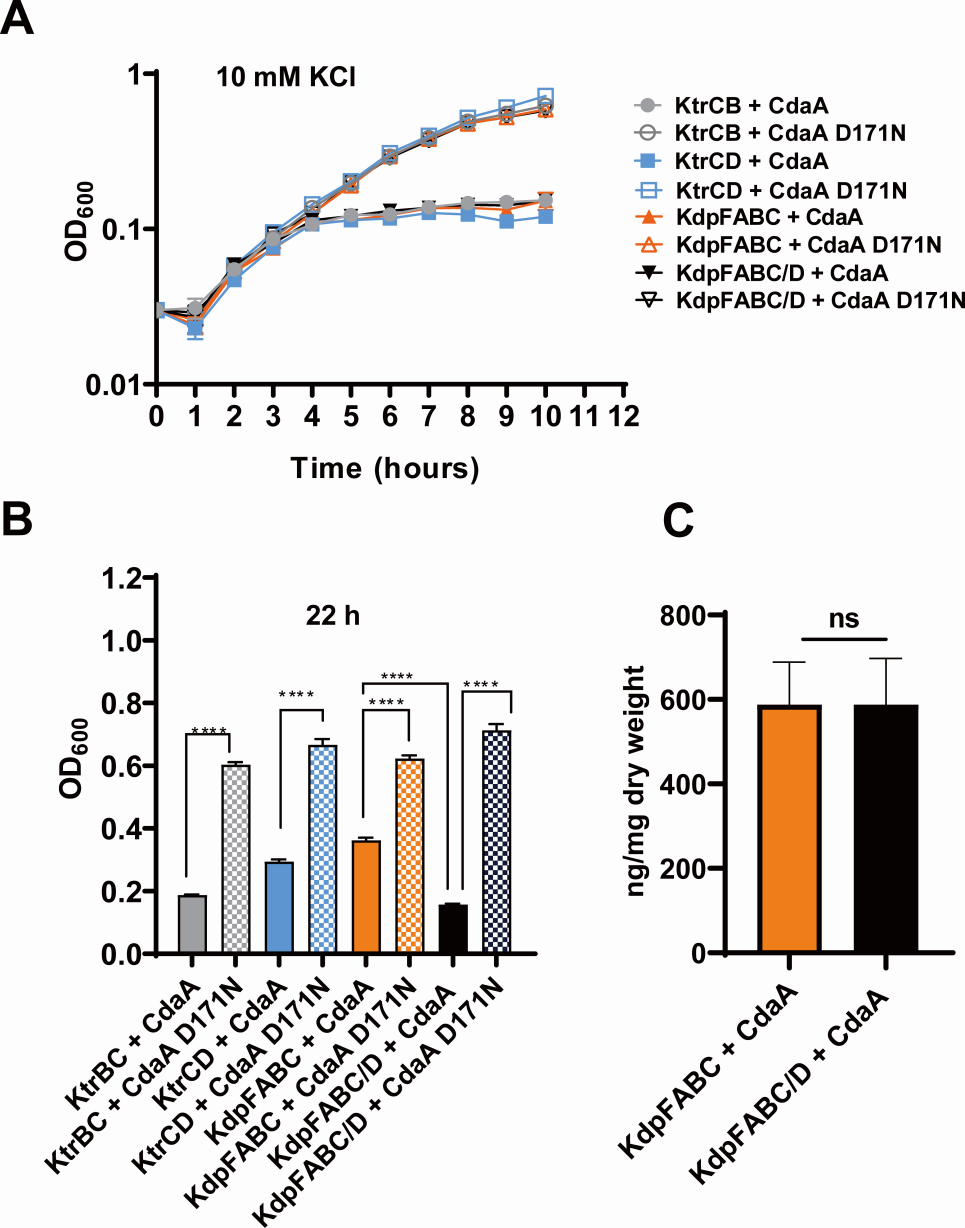
Inhibition of KtrCB/CD and KdpFABC transport activity by c-di-AMP. (**A**) *E. coli* LB2003 carrying relevant plasmids were cultivated in M9 medium supplemented with 80 µM IPTG for KtrCB/CD and KdpFABC induction and 0.005% arabinose for the induction of the *cdaA* alleles. The different potassium transport systems (KtrCB/CD/FABC) are expressed via pQE60 and CdaA/CdaA D171N via pBAD33. (**B**) Optical density (OD)_600_ of 22 h growth in M9 medium. Means and standard errors of the means (SEMs) are shown. *n* = 3. ^****^P < 0.0001 by two-tailed Student’s *t*-test. The different potassium transport systems (KtrCB/CD/ABC) are expressed via pQE60 and CdaA/CdaA D171N via pBAD33. (**C**) Intracellular c-di-AMP concentration. Means and SEMs are shown; *n* = 3. ^*^P < 0.05. All data were analyzed by two-tailed Student’s *t*-test.

The specific interaction of KdpD and KtrC with c-di-AMP indicates a functional role for c-di-AMP in controlling KdpFABC/D and KtrCB/D activity. To examine whether c-di-AMP affects the activity of KdpFABC/D, KtrCB, and KtrCD, we established a co-expression system using the *E. coli* strain LB2003, which is deficient in the Kdp*,* Kup, and Trk potassium uptake systems. This K^+^ uptake-deficient *E. coli* strain can only grow at low potassium concentrations when synthesizing a single potassium transporter (40). *E. coli* lacks c-di-AMP-synthesizing enzymes and is unable to produce the second messenger. Plasmid pQE60 which encodes proteins such as ktrCB*,* KtrCD*,* KdpFABC*,* or KdpFABC/D, was used for the isopropyl-β-D-1-thiogalactopyranoside (IPTG)-dependent expression of *ktr/kdp* genes, wherea*s cdaA* from *L. monocygogenes* (*cdaA*^lmo^) and inactivated *cdaA*^lmoD171N^ were expressed from plasmids pBAD33-*cdaA* and pBAD33-*cdaA D171N*, respectively (41). The growth phenotype upon the co-expression of CdaA^Lmo^ and *kdpD/ktrC* genes reflects the effect of c-di-AMP binding on KdpD/KtrC. The growth of bacteria synthesizing KtrCB, KtrCD, and KdpFABC/D was severely impaired when the active diadenylate cyclase CdaA was co-produced, indicating that c-di-AMP inhibits the transporter (Fig. 2A). In the absence of functional CdaA, normal cell growth under a high K^+^ environment was restored (Fig. 2A). Moreover, growth of bacteria synthesizing KdpFABC was also reduced 1.72-fold when the active DAC CdaA was co-produced and to a lesser extent than KdpFABC/D (4.54-fold) (Fig. 2B), indicating that c-di-AMP may directly affect the expression of KdpFABC. How does c-di-AMP inhibit KdpFABC in *E. coli*? Although the *kdpA/B/C* is absent in *E. coli* LB2003, both *kdpD* and *kdpE* are amplified in the strain (Fig. S1 at http://dx.doi.org/10.6084/m9.figshare.25669899). We hypothesized that c-di-AMP-bound KdpD from *E. coli* would also down-regulate the expression of KdpFABC. Therefore, the potassium uptake systems KtrCB/D and KdpFABC from *B. anthracis* are both inhibited by c-di-AMP. To rule out the possibility of c-di-AMP affecting bacteria growth, the intracellular c-di-AMP concentrations were measured in CdaA+KdpFABC and CdaA+KdpFABC/D (Fig. 2C). The fact that there was no change in intracellular c-di-AMP concentration suggested that KdpD from *B. anthracis* is functional and the KdpFABC is more severely suppressed by c-di-AMP in the presence of KdpD*_B. anthracis_*.

**Fig 3:**
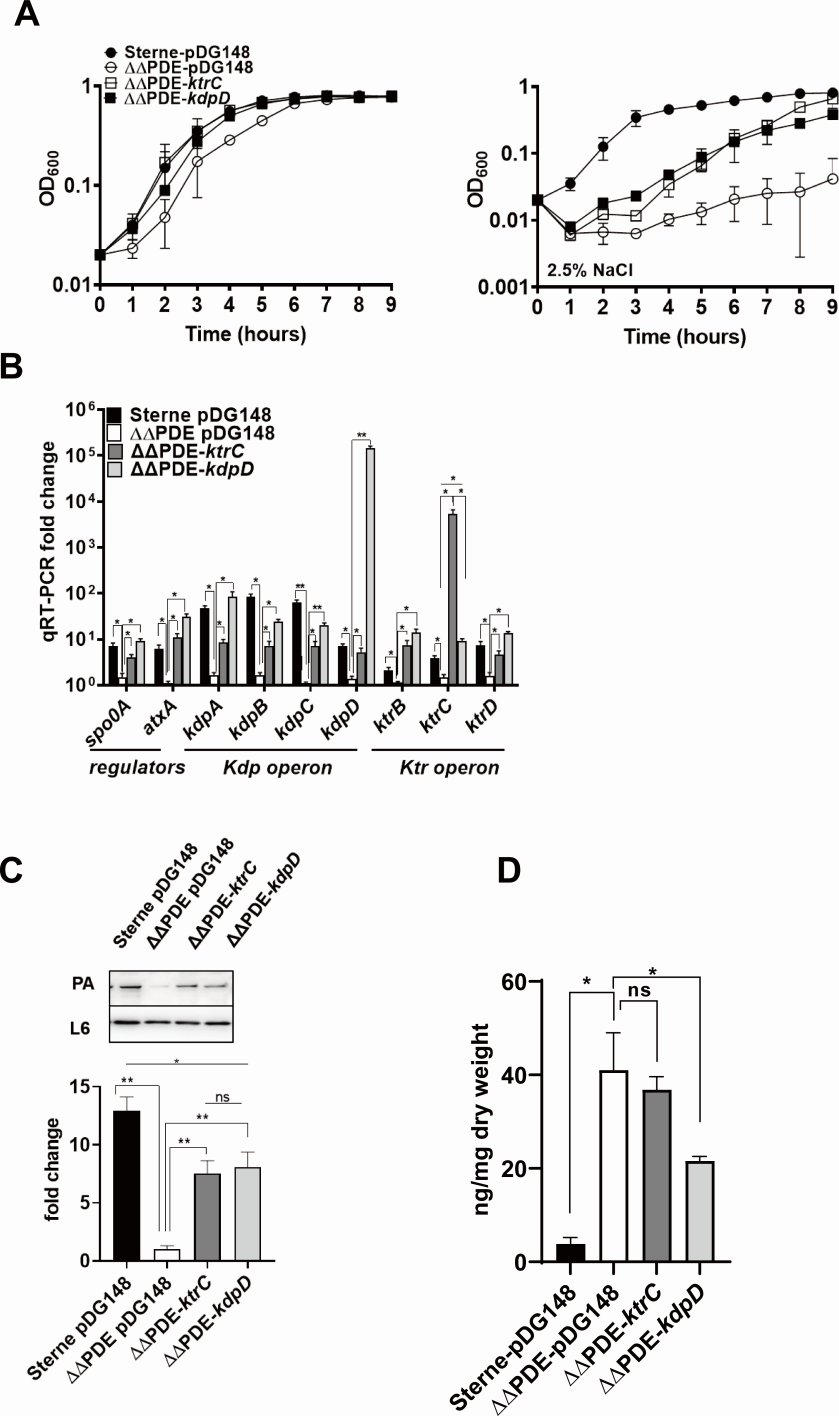
Overexpression of KdpD and KtrC restored anthrax toxin expression and resistance to osmotic stress in the c-di-AMP accumulated strain. (**A**) Representative growth kinetics of *B. anthracis*, ΔΔPDE, ΔΔPDE-*ktrC,* and ΔΔPDE-*kdpD* in BHI broth without or with 2.5% NaCl. OD_600_ at the indicated time points was measured. (**B**) c-di-AMP accumulation repressed KtrCB/CD and KdpFABC. qRT-PCR was used to measure the transcript abundance of potassium uptake genes. RNA was isolated from bacteria grown to the stationary phase in BHI (0.8% NaHCO_3_). Expression levels of each gene were normalized to *tufA*. Means and SEMs are shown; *n* = 3. ^*^P < 0.05; ^**^P < 0.01. All data were analyzed by using one-way analysis of variance followed by Tukey’s post-test analysis. (**C**) Overexpression of KdpD enhanced the expression of the anthrax toxin. (Up) The stationary phase strains were subjected to immunoblotting with antisera raised against PA (the toxin subunit) and L6 (the loading control). The blots are representative of three replicates. (Down) Gray values of the bands. Means and SEMs are shown; *n* = 3. ^*^P < 0.05 by two-tailed Student’s test. (**D**) Intracellular c-di-AMP concentration. Means and SEMs) are shown; *n* = 3. ^*^P < 0.05. All data were analyzed by using one-way analysis of variance followed by Tukey’s post-test analysis.

Impairment of KtrCB/D and KdpFABC systems by c-di-AMP in *E. coli* LB2003 prompted us to examine the impact of c-di-AMP on KtrCB/D and KdpFABC expression. Previous research showed that elevated levels of c-di-AMP result in increased susceptibility to osmotic stress. The ΔΔPDE mutants (37) were unable to grow even at a mild salt concentration. A growth assay showed that the expression of KtrC and KdpD partially restored the growth defects of ΔΔPDE in the brain heart infusion (BHI) (2.5% NaCl) medium (Fig. 3A). Quantitative reverse transcription (qRT)-PCR results of the c-di-AMP accumulated strain (ΔΔPDE) revealed that the transcriptional levels of the Kdp system were reduced 5- to 56-fold, including *kdpA*, *kdpB*, *kdpC*, and *kdpD* (Fig. 3B). The Ktr system, including *ktrC*, *ktrB*, and *ktrD*, also attenuated 1.9- to 4.7-fold in c-di-AMP accumulated strain (Fig. 3B). After transforming pDG148-*kdpD* or pDG148-*ktrC* into ΔΔPDE, we observed that the overexpression of *kdpD* or *ktrC* promoted the expression of *kdpA/B/C* and *ktrB/C/D* (Fig. 3B). The overexpression of KdpD or KtrC also restored the anthrax toxin deficiency in the c-di-AMP accumulated strain (Fig. 3C). As the c-di-AMP receptor, the overexpression of KdpD indeed decreased the intracellular c-di-AMP whereas the overexpression of KtrC did not affect the c-di-AMP concentration (Fig. 3D). These results suggested that upregulating potassium uptake instead of intracellular c-di-AMP change is critical for anthrax toxin regulation.

**Fig 4:**
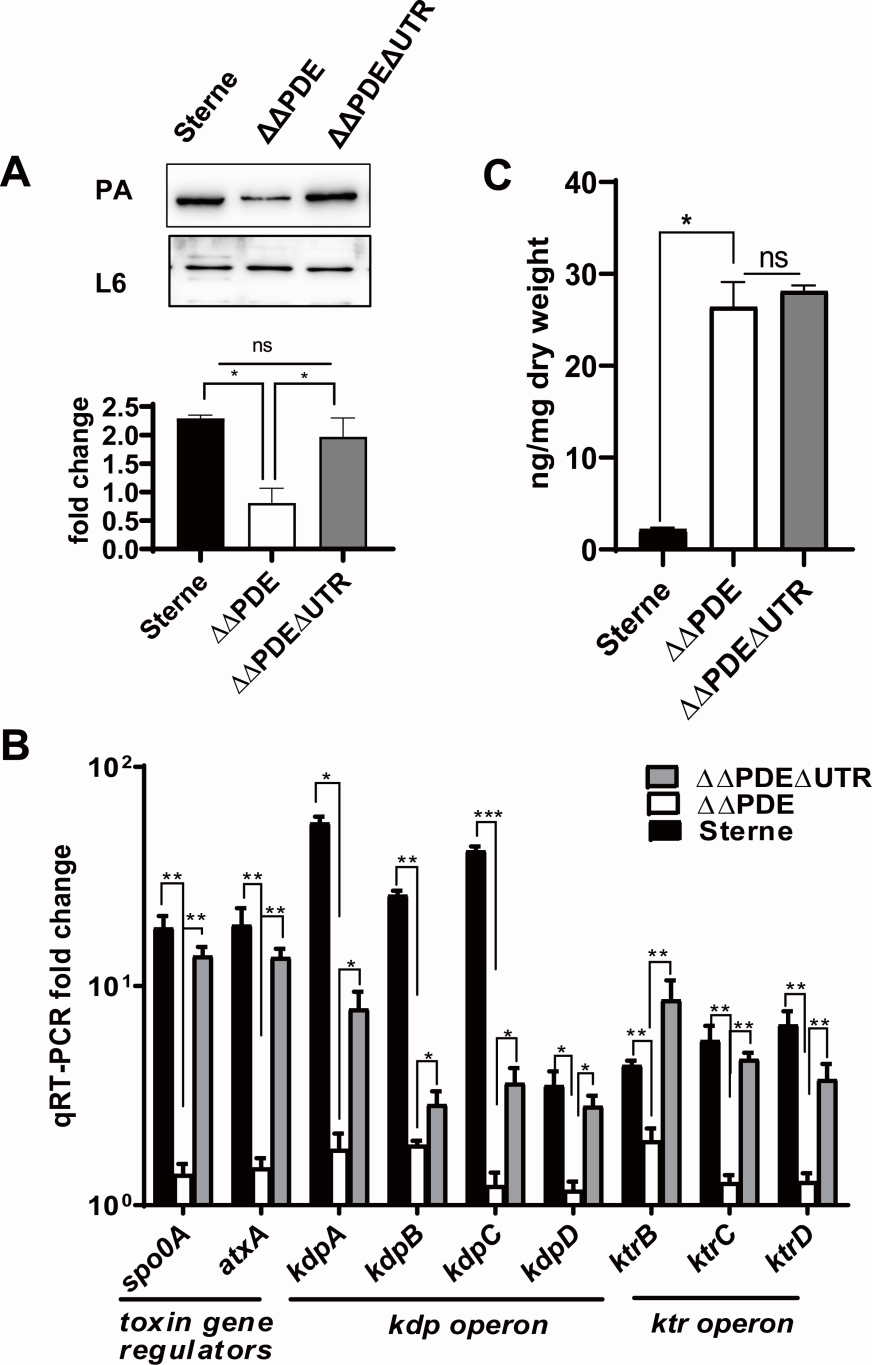
Deletion of UTR (the upstream of *kdp*) stimulated anthrax toxin expression in the c-di-AMP accumulated strain. (**A**) Deletion of UTR restored anthrax toxin PA expression. (Up) The stationary strains were subjected to immunoblotting with antisera raised against PA (the toxin subunit) and L6 (the loading control). The blots are representative of three replicates. (Down) Gray values of the bands. Means and SEMs are shown; *n* = 3. ^*^P < 0.05 by using one-way analysis of variance followed by Tukey’s post-test analysis. (**B**) qRT-PCR was used to measure transcript abundance of *kdp* operon genes, toxin gene regulator, and *spo0A* kinase genes. RNA was isolated from bacteria grown to the stationary phase in BHI (0.8% NaHCO_3_). Expression levels of each gene were normalized to *tufA*. Means and SEMs are shown; *n* = 4. ^#^*P* < 0.1; ^*^P < 0.05; ^***^P < 0.001. All data were analyzed by using one-way analysis of variance followed by Tukey’s post-test analysis. (**C**) Intracellular c-di-AMP concentration. Means and SEMs) are shown; *n* = 3. ^*^P < 0.05. All data were analyzed by using one-way analysis of variance followed by Tukey’s post-test analysis.

Wang et al. (23) found a c-di-AMP riboswitch *ydaO* sequence upstream of the *kdp* transcript in *B. thuringiensis*. The riboswitch encoding region was present in the 5′-untranslated region (UTR) encoding region (147–346 bp upstream of the start codon). c-di-AMP represses *kdp* operon transcription by enhancing the transcriptional termination of *ydaO*. A BLAST N search revealed that the same c-di-AMP riboswitch that regulated the *kdp* operon also existed in *B. anthracis* (147–346 bp upstream of the start codon). The sequence of the riboswitch was identical to *B. thuringiensis*. The role of the c-di-AMP riboswitch in regulation was assessed by constructing a ΔUTRΔΔPDE strain where the c-di-AMP riboswitch encoding region from the *B. anthracis* Sterne genome was deleted. Immunoblotting results demonstrated that anthrax toxin expression was restored in the ΔUTRΔΔPDE strain (Fig. 4A). qRT-PCR results suggested that the transcriptional level of *kdpD* increased by approximately threefold upon deletion of UTR (Fig. 4B). The transcriptional levels of the Kdp system were increased 1.5- to 4.4-fold, including *kdpA*, *kdpB*, and *kdpC*. The transcriptional levels of *ktrB, ktrC*, and *ktrD* were significantly enhanced and rebound to parental strain levels upon the deletion of UTR. For the anthrax toxin regulator, the transcriptional level of *atxA* increased by ninefold and rebounded to the parental strain levels (Fig. 4B). The transcriptional level of *spo0A* increased by 10-fold in the ΔUTRΔΔPDE strain when compared with the ΔΔPDE strain. The intracellular c-di-AMP levels were not significantly changed upon deletion of the UTR (Fig. 4C). The results suggested that the inactivation of *ydaO* in *B. anthracis* upregulated the expression of *kdp* and *ktr* operon, which in turn restored the production of the anthrax toxin in the c-di-AMP accumulated strains.

### Inactivation of potassium uptake genes affects osmotic resistance and anthrax toxin expression in *B. anthracis*

**Fig 5:**
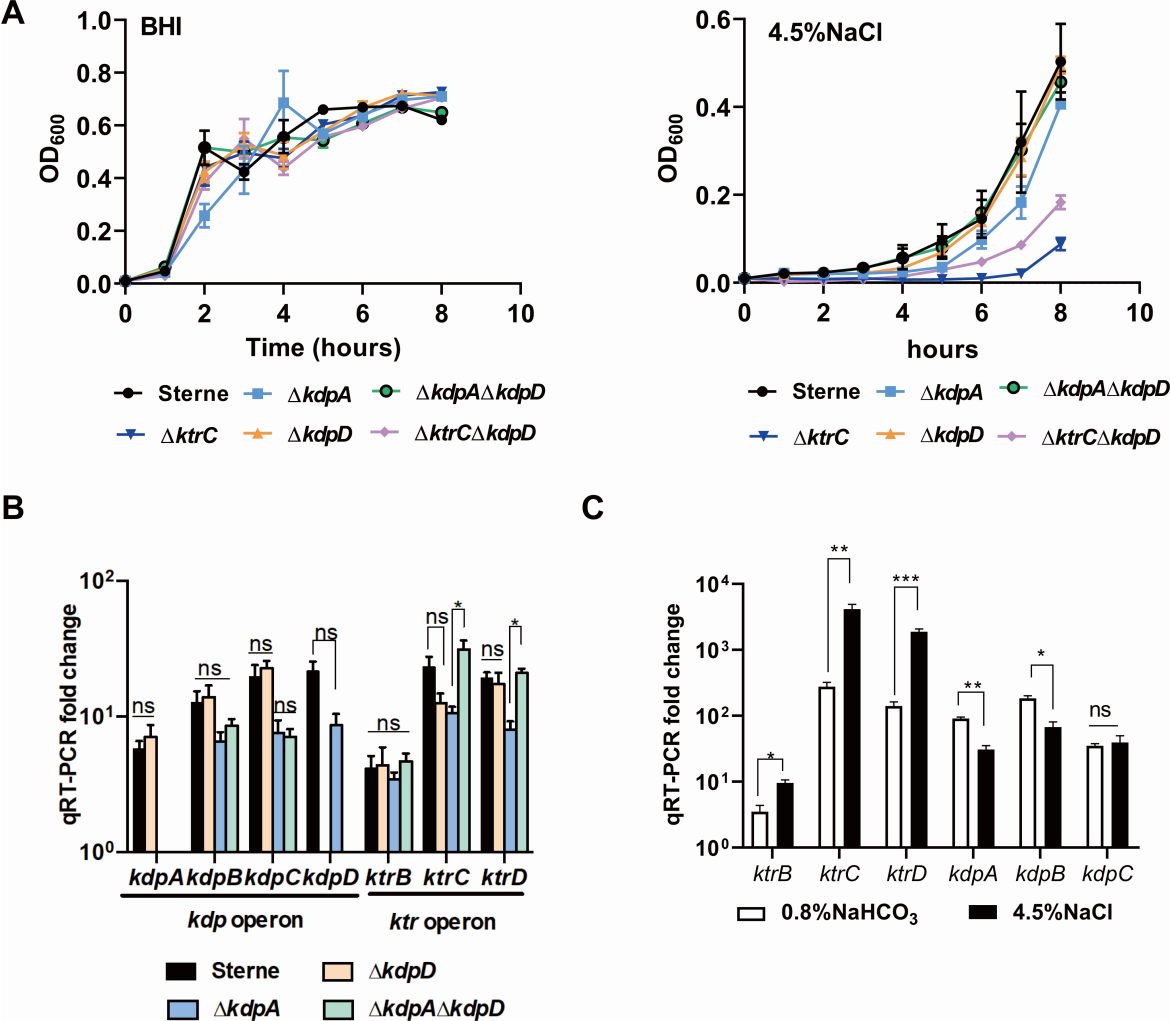
Inactivation potassium uptake genes affect osmotic resistance in *B. anthracis*. (**A**) Representative growth kinetics of *B. anthracis* and potassium uptake gene mutants in BHI or BHI broth supplemented with 4.5% NaCl. OD_600_ at the indicated time points was measured. (**B**) qRT-PCR was used to measure the transcript abundance of *kdp* operon and *ktr* operon genes. RNA was isolated from bacteria grown to the stationary phase in BHI (4.5% NaCl). Expression levels of each gene were normalized to *tufA*. Means and SEMs are shown; *n* = 4. ^*^P < 0.05. All data were analyzed by using one-way analysis of variance followed by Tukey’s post-test analysis. (**C**) qRT-PCR was used to measure the transcript abundance of *kdp* operon and *ktr* operon genes in Sterne strain. RNA was isolated from bacteria grown to the stationary phase in BHI (0.8% NaHCO_3_ or 4.5% NaCl). Expression levels of each gene were normalized to tufa. Means and SEMs are shown; *n* = 4. ^*^P < 0.05. All data were analyzed by two-tailed Student’s *t*-test.

The function of the potassium uptake system in the *B. anthracis* strain was examined by creating a series of mutants with defects in potassium transport components, Δ*kdpA*, Δ*kdpD*, Δ*ktrC*, Δ*ktrC*Δ*kdpD,* and Δ*kdpA*Δ*kdpD.* The growth curves of these mutants were indistinguishable when compared with the growth of the parental strain in the BHI medium. We also examined cell growth in a salt medium (4.5% NaCl) and observed that the growth of Δ*ktrC*Δ*kdpD* and Δ*ktrC* was inhibited in the BHI medium (4.5% NaCl) (Fig. 5A). The transcription levels of *kdpA/B/C* and *ktrB/C/D* were not significantly changed in Δ*kdpD* under 4.5% NaCl (Fig. 5B). qRT-PCR results showed that *ktr* operon was induced 2.7- to 15-fold under 4.5% NaCl treatment, whereas *kdpA/B* was decreased (Fig. 5C). These results suggest that Δ*ktrC* is sensitive to NaCl due to the strongly induced *ktr* operon.

**Fig 6:**
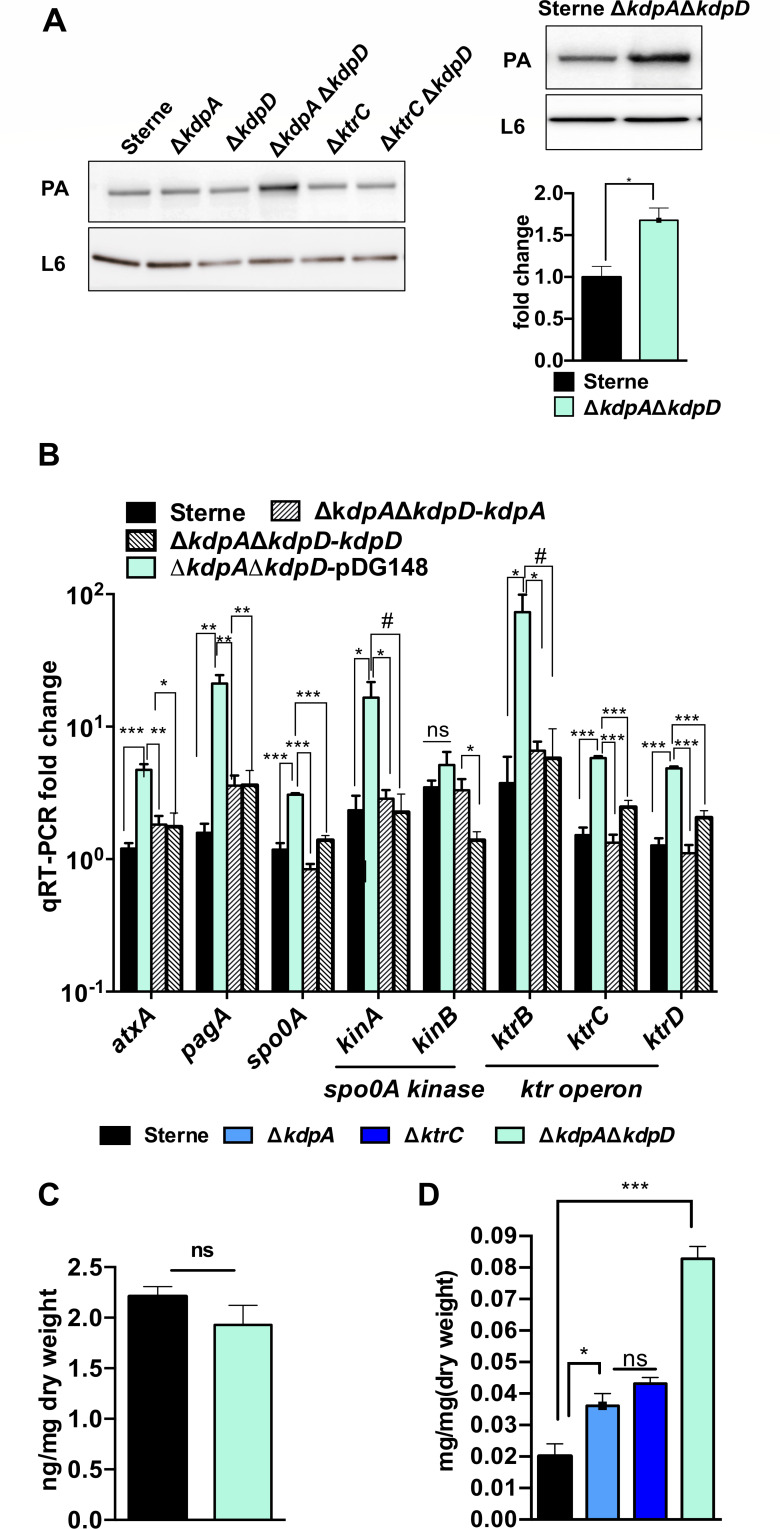
Inactivation of *kdpA* and *kdpD* enhanced the expression of the anthrax toxin and *ktrCD* in BHI (0.8% NaHCO_3_). (**A**) Inactivation of *kdpA* and *kdpD* promoted anthrax toxin expression. (Left) The strains were subjected to immunoblotting with antisera raised against PA and L6. (Right up) The stationary phase strains were subjected to immunoblotting with antisera raised against PA (the toxin subunit) and L6 (the loading control). The blots are representative of three replicates. (Down) Gray values of the bands. Means and SEMs are shown; *n* = 3. ^*^P < 0.05 by two-tailed Student’s test. (**B**) Inactivation of *kdpA* and *kdpD* stimulated *ktrBCD* expression. RNA was isolated from bacteria grown to the stationary phase in BHI (0.8% NaHCO_3_). Expression levels of each gene were normalized to *tufA*. Means and SEMs are shown; n = 4. ^*^P < 0.05; ^**^P < 0.01; ^***^P < 0.001. All data were analyzed by using one-way analysis of variance followed by Tukey’s post-test analysis. (**C**) Intracellular c-di-AMP concentration. Means and SEMs) are shown; *n* = 3. All data were analyzed by two-tailed Student’s *t*-test. (**D**) Intracellular potassium concentration. Means and SEMs) are shown; *n* = 3. ^*^P < 0.05; ^***^P < 0.001. All data were analyzed by two-tailed Student’s *t*-test.

Immunoblot results demonstrated that anthrax toxin expression was enhanced in the Δ*kdpA*Δ*kdpD* strain under non-stress conditions (Fig. 6A). qRT-PCR results showed that *ktrB*, *ktrC,* and *ktrD* expression increased significantly in the Δ*kdpA*Δ*kdpD* mutant strain (approximately 5- to 20-fold), suggesting that the inactivation of *kdpA* and *kdpD* stimulates another potassium uptake system, the Ktr operon, which was consistent to the result in BHI (0.8% NaHCO_3_) (Fig. 6B). The intracellular c-di-AMP concentration was measured in Sterne and Δ*kdpA*Δ*kdpD,* and the results revealed no significant differences between them, demonstrating that the alteration in potassium absorption alone would affect anthrax toxin expression (Fig. 6C). The intracellular potassium concentration was measured among Sterne, Δ*kdpA*, Δ*ktrC*, and Δ*kdpA*Δ*kdpD*. The results showed that the intracellular potassium concentration in Δ*kdpA*Δ*kdpD* increased by fourfold compared with the parental strain, whereas Δ*kdpA or* Δ*ktrC* increased by twofold (Fig. 6D). Therefore, the inactivation of both *kdpA* and *kdpD* promoted potassium uptake and anthrax toxin expression in *B. anthracis.* We further examined *kdpD* expression by comparing Sterne versus Δ*kdpD* strains and ΔΔPDE versus ΔΔPDEΔ*kdpD* strains. The results also showed that the inactivation of *kdpD* promoted *kdpB/C* and *ktrD* (Fig. S2 at http://dx.doi.org/10.6084/m9.figshare.25669923).

## DISCUSSION

**Fig 7:**
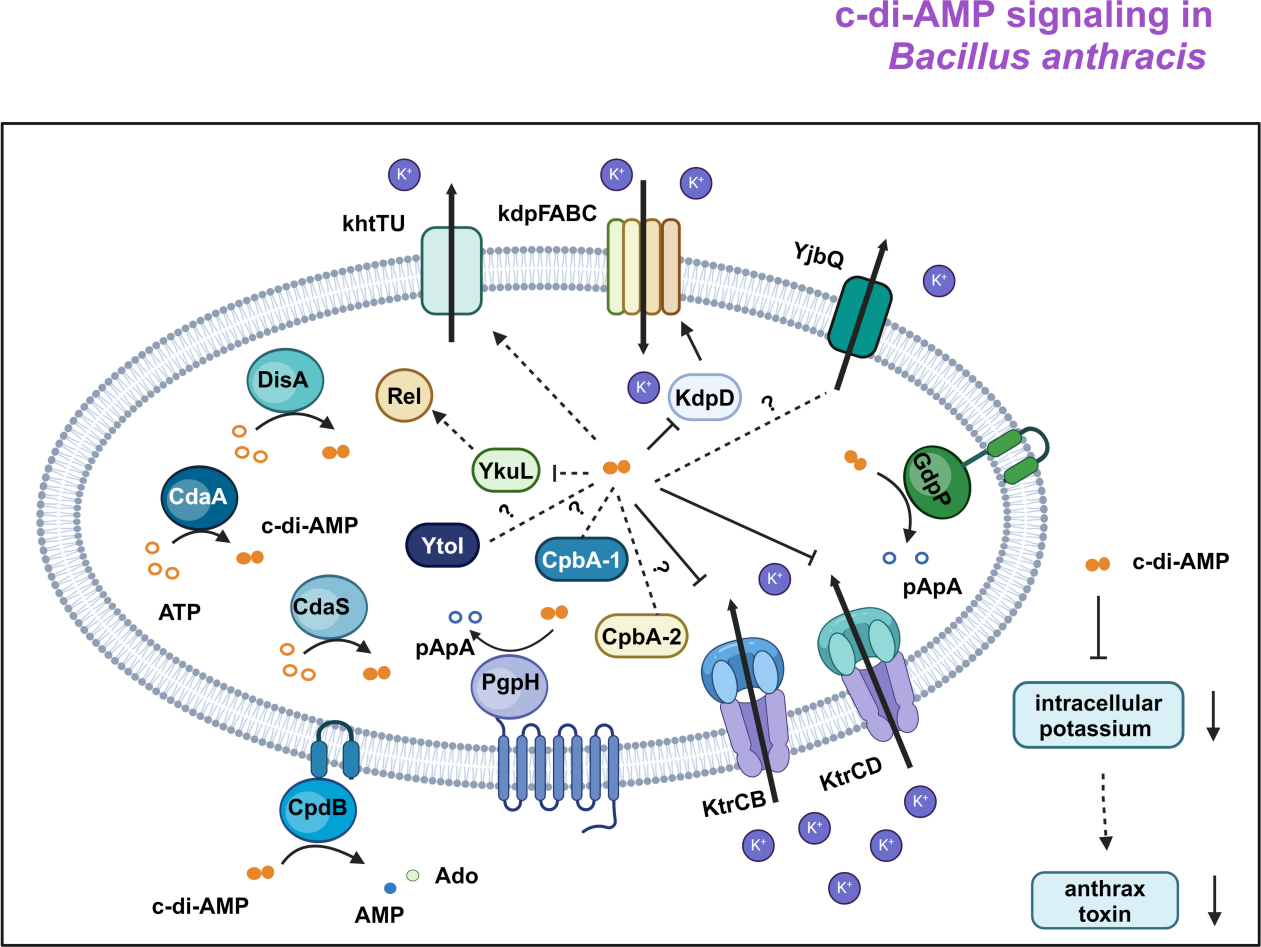
c-di-AMP signaling in *Bacillus anthracis*. The second messenger c-di-AMP is synthesized by the diadenylate cyclase DisA, CdaA, and CdaS through two molecules of ATP. Degradation of c-di-AMP is facilitated by the phosphodiesterase GdpP, PgpH, and CpdB. c-di-AMP binds to signal transduction proteins and controls the expression and activity of several potassium transporters by binding to mRNA riboswitches and proteins, respectively. c-di-AMP accumulation would down-regulate intracellular potassium and reduce anthrax toxin expression in *B. anthracis.* Solid arrows indicate activation; solid lines without arrowheads indicate inhibition; dash lines indicate potential receptors; dash arrows indicate potential activation; open brown circles indicate ATP; close brown circles indicate c-di-AMP; open blue circles indicate pApA; close blue circles indicate AMP; close light green circles indicate Ado.

Our previous study suggested that c-di-AMP accumulation impaired *B. anthracis* virulence and toxin expression. c-di-AMP mainly regulates osmolyte homeostasis in bacteria and archaea (42). In *B. subtilis*, c-di-AMP modulates the uptake of potassium ions via different potassium transport systems. To analyze how c-di-AMP inhibits toxin expression, we did pull-down and DraCALA assays to screen c-di-AMP receptors. Nine c-di-AMP receptors were identified, and five of them were involved in potassium uptake. Therefore, we proposed that c-di-AMP suppress anthrax toxin expression by regulating potassium uptake. Overaccumulation of c-di-AMP reduces the intracellular potassium pool in bacteria (25). However, it is unclear whether c-di-AMP accumulation or the decreased potassium uptake impaired toxin expression. Our results demonstrated that c-di-AMP accumulation affects anthrax toxin expression by suppressing the potassium uptake system (Fig. 7). Overexpression of the c-di-AMP receptor KdpD and KtrC enhanced the Ktr and Kdp potassium uptake system and restored the expression of the anthrax toxin. Deleting the 5′-UTR regulatory region of the *kdp* operon also increased both the potassium uptake system (Ktr and Kdp) expression and the production of the anthrax toxin. The overexpression of KtrC or inactivation of *ydaO* does not alter the intracellular c-di-AMP concentration, which suggested that reduced potassium uptake might be the reason for the regulation of anthrax toxin expression. Overaccumulation of c-di-AMP reduces the intracellular potassium pool in bacteria (25). Potassium inhibits KinC-dependent biofilm formation and stimulates Spo0A-phosphorelay histidine kinase KinB (43). In *B. anthracis*, phosphorylation of Spo0A promotes anthrax toxin gene expression by repressing AbrB expression (44). Further studies are required to determine whether potassium absorption system inhibition affects Spo0A phosphorylation. However, we cannot make a conclusion that PDE mutant is defective for anthrax toxin production because of a deficiency in potassium. Another possibility for increasing potassium uptake restoring anthrax toxin might be compensatory. Further studies are required to analyze whether potassium is required for anthrax toxin production. *B. thuringiensis* possesses at least three types of K^+^ uptake transporters, including the Trk system, Kdp system, and KimA protein, whereas *B. subtilis* possesses KtrAB, KtrCD, and KimA (23). The potassium uptake systems in *B. anthracis* differ from *B. subtilis* and *B. thuringiensis.* K^+^ uptake in *B. anthracis* is mediated by two transport systems, Ktr and Kdp. For the Ktr systems, K^+^ uptake probably occurs with the symport of sodium ions. The membrane components KtrB and KtrD probably work in tandem with the gating component KtrC as the Ktr potassium uptake components in *S. aureus* (45). Under high osmolarity and K^+^-limiting circumstances, the Ktr system is crucial for bacterial growth (33). c-di-AMP binds directly to KtrC, the membrane-associated component of the potassium transporter KtrC-KtrD and KtrC-KtrB, and prevents potassium uptake by *B. anthracis*. The Kdp system is required for bacterial growth in defined media under K^+^-limiting conditions. The main complex KdpFABC is composed of a P-type ATPase KdpB (16, 46) with a channel-like subunit (KdpA) (47), the lipid-like stabilizer KdpF (48), and the unknown functional component KdpC. At low K^+^ concentrations, the transcriptional level of KdpFABC is activated by the two-component system KdpD/KdpE (49). KdpD is located downstream of KdpC, which consists of an N-terminal domain (NTD) and a C-terminal domain (CTD). The NTD is necessary for the highest level of *kdpFABC* expression, whereas the CTD is critical for phosphotransfer reactions (50). After stimulation, KdpE acquires the phosphoryl group from KdpD and promotes the transcription of the *kdpFABC* operon. In *B. anthracis,* KdpD only contains the N-terminal sensor region of KdpD but lacks the C-terminal histidine kinase region. Although lacking *kdpE* in the *B. anthracis* genome, our results showed that c-di-AMP binds to KdpD, which down-regulates the expression of KdpFABC significantly. How c-di-AMP-bound KdpD repressed KdpFABC in the absence of KdpE requires further analysis. KdpFABC and KtrCB/D are probably the only two potassium uptake systems in *B. anthracis* because we cannot generate the Δ*kdpA*Δ*ktrC* double mutant strain.

Based on our result, the Ktr system appears to play a dominant role in moderate osmotic stress resistance in *B. anthracis*. Growth of Δ*ktrC* was impaired under 4.5% NaCl treatment, whereas growth of Δ*kdpA* was not affected. The Δ*ktrC* is sensitive to 4.5% NaCl probably because *ktrC/D* is strongly induced (13–15-fold) under this condition. Furthermore, our results showed that the deletion and overexpression of *kdpD* increased *ktr* operon expression. In c-di-AMP accumulated ΔΔPDE strain, *kdpD* over-expression increased *ktr* operon expression because it decreased the intracellular c-di-AMP, thus releasing the suppression of the *ktr* operon. The enhanced *ktr* potassium uptake system compensates for the repression of the *kdp* system in *B. anthracis*. Probably, inactivating one of them would stimulate the other. In the *kdpA* repressed strain such as ΔΔPDE and Δ*kdpA, ktr* operon was induced with the absence of *kdpD.* c-di-AMP-bound KdpD might repress *ktr* operon with mechanisms requiring further analysis.

## MATERIALS AND METHODS

### Bacterial strains, plasmids, and growth conditions

The bacterial strains and plasmids are listed in Table S1 at http://dx.doi.org/10.6084/m9.figshare.25669950. The bacterial strains were grown in either Luria broth (LB) or BHI broth (Oxoid) under aerobic conditions at 37°C.

The mutants were constructed by markerless gene deletion methods, as described previously (13, 51). The deletion of these genes was confirmed by DNA sequencing using the primers. The PCR confirmation results from the mutant and Sterne strain are shown in Fig. S3 at http://dx.doi.org/10.6084/m9.figshare.25669938.

### Plasmid construction

The *kdpA*, *ktrC,* and *kdpD* alleles with the Shine-Dalgarno (SD) sequence were amplified using chromosomal DNA of *B. anthracis* Sterne as the template, and the fragments were cloned between specific restriction sites SphI and HindIII in pDG148. LacIQ was synthesized and inserted into the XbaI cloning site of pQE60 to create the IPTG inducible expression plasmid. The genes encoding putative potassium transporters were then introduced into the vector pQE60. For the construction of pQE60*-ktrC*, the *ktrC* gene was amplified using oligonucleotide pair *pQE60-ktrCF* and *pQE60-ktrCR* and ligated to pQE60 between EcoRI and HindIII. The *ktrB* and *ktrD* genes were amplified using oligonucleotide pairs *ktrB*pQE60F/*ktrB*pQE60R and *ktrD*pQE60F/*ktrD*pQE60R, respectively, and fused to pQE60-*ktrC* by the infusion assay to create pQE60*-ktrCD* and pQE60*-ktrCB*. To construct pBAD33 *cdaA*^lmo^, the *cdaA* gene was amplified from *L. monocytogenes* EGD-e chromosomal DNA using oligonucleotides pBad33 *cdaA*F and pBad33 *cdaA*R and cloned between specific restriction sites XbaI and HindIII in pBAD33. pBAD33-*cdaA*_lmoD171N_ was constructed using pBAD33-*cdaA* as the template, and overlapping PCR was performed with the oligonucleotides *cdaA* D171NF and *cdaA* D171NR. *kdpD* and 13 other potential c-di-AMP receptor genes were amplified using Sterne chromosomal DNA as the template and cloned between the NcoI and XhoI sites of the expression plasmid pET28a. The plasmid was then transformed into *E.coli* BL21 (DE3) cells. The DisA-6×His fusion protein was purified as described previously (13). All primers are listed in Table S2 at http://dx.doi.org/10.6084/m9.figshare.25669959.

### Differential radial capillary action of ligand assay

The 14 selected genes were cloned into the expression vector pET28a and expressed as C-terminal His-tag fusion proteins (Table 1). The expression vectors were transformed into *E. coli* BL21 cells, and induced protein expression was detected and confirmed, except for YugO, by immunoblotting. The protein patterns of the expression strains were analyzed by SDS-PAGE. This analysis enabled confirmation that genes were expressed after being induced with 0.8 mM IPTG. Cells were collected by centrifugation; re-suspended in a c-di-AMP binding buffer (10 mM Tris base, 100 mM NaCl, and 5 mM MgCl_2_, pH 8.0) containing 10 µg/mL DNase, 250 µg/mL lysozyme, and 1 µM PMSF; incubated for 0.5 h; and subjected to two freeze–thaw cycles (16, 24, 52, 53). Cell lysates were subsequently stored at –80°C. After thawing, 20 µL *E. coli* whole lysates were mixed with radioisotope-labeled c-di-AMP and incubated for 20 min, and 2 µL of these mixtures was spotted onto nitrocellulose membranes (PALL) using a multi-channel pipette. Membranes were allowed to dry after spotting. The radioactivity intensity was detected by a phosphor screen (PerkinElmer) and Cyclone Plus (PerkinElmer).

**TABLE 1:** Expression plasmids used in the DraCALA assay

Plasmid	Accession number	Function	Domain
pET28a-*ktrC*	**BA_4191 ^*a*^**	Peripheral membrane component K^+^ transporter	RCK_C
pET28a-*khtT*	**BA_5287 ^*a*^**	K^+^/H^+^ antiporter	RCK_C
pET28a-*khtT-2*	**BA_4994 ^*a*^**	K^+^/H^+^ antiporter	RCK_C
pET28a-*yjbQ*	**BA_0699 ^*a*^**	Potassium exporter	RCK_C
pET28a-*yugo*	BA_5182	Unknown function	RCK_C
pET28a-*kdpD*	**BA_0742 ^*a*^**	Putative sensor protein	
pET28a-*prov2*	BA_2786	Glycine betaine/L-proline ABC transporter, ATP-binding protein	CBS
pET28a-*ykuL*	**BA_4196 ^*a*^**	Unknown function	CBS
pET28a-*yrkA*	BA_3422	Unknown function	CBS
pET28a-*acuB*	BA_4917	Unknown function	CBS
pET28a-*ccpN*	BA_4521	Potential transcriptional repressor	CBS
pET28a-*ytoI*	**BA-4858 ^*a*^**	Unknown function	CBS
pET28a-*cbpA-1*	**BA_2061 ^*a*^**	Unknown function	CBS
pET28a-*cbpA-2*	**BA_1348 ^*a*^**	Unknown function	CBS

^*a*^ The bold values mean the positive candidates in the DraCALA assay.

^32^P-c-di-AMP was prepared using 55 nM α^32^P-ATP incubated with 20 µM DisA from *B. anthracis* at 30°C overnight. Five units of calf intestine alkaline phosphatase (Fermentas) were added to the mixture for 1 h. After incubating at 95°C for 10 min, the denatured proteins were removed by centrifugation, and the supernatant was stored. The competition assays were performed with indicated concentrations of unlabeled ATP and c-di-AMP (Sigma).

### Growth curves in minimal salt medium

The potassium transporter-deficient *E. coli* strain LB2003 was co-transformed with the plasmid pQE60 derivatives (pQE60-*ktrCB,* pQE60-*ktrCD*, pQE60-*kdpFABC*, and pQE60-*kdpFABCD*) and the pBAD33 derivatives (pBAD33-*cdaA* or pBAD33-*cdaA D171N*) on LB plates containing ampicillin and chloramphenicol. Single colonies were used to inoculate 4 mL M9 medium (35 mM KCl) containing 0.2% (wt/vol) glycerol and 0.02% (wt/vol) glucose (20, 27). The cultures were incubated overnight at 37°C and used to re-inoculate the same medium (without glucose) to an optical density (OD)_600_ = 0.1. Then, the cells were washed and re-suspended in M9 medium (10 mM KCl) supplemented with 0.2% (wt/vol) glycerol, 0.005% arabinose, and 80 µM IPTG. Growth was monitored using an Epoch 2 microplate spectrophotometer (BioTek Instruments).

### Quantitative real-time PCR

The *B. anthracis* Sterne strain and deletion mutants were grown overnight in BHI (0.8% NaHCO_3_). Total mRNA was extracted with the Bacteria Extraction kit (R403-01; Vazyme Biotech Co., Ltd.) and reverse-transcribed using the QuantiTect Reverse Transcription kit (Qiagen, Valencia, CA). With a CFX96 real-time PCR detection device, cDNAs were used as the templates for real-time qRT-PCR investigation of selected genes (Bio-Rad, California). 2× Universal SYBR Green Fast qPCR Mix (ABclonal) was applied for all RT-PCRs. Primers are listed in Table S2. *tufA* was used as the internal control. The amplification efficiency was 0.9–0.99.

### Assay of anthrax toxin in supernatants

Sterne strain and deletion mutants were inoculated in 5 mL LB broth and incubated for 16 h at 37°C. Secondary inoculation (0.1%) was performed in 5 mL BHI (Oxoid) (0.8% NaHCO_3_), and the cultures were incubated at 37°C for 18 h. The protein samples were collected and prepared as described previously (37). Membranes were blotted with antibodies against PA (1:1,000 dilution, Abcam catalog # ab13808, RRID: AB_300652) and L6 (provided by Chun-Jie Liu, Beijing Institute of Biotechnology: a ribosomal protein in the cytoplasm, 1:1,000 dilution) and then with the anti-rabbit IgG horseradish peroxidase-conjugated secondary antibody (1:5,000 dilution).

### Salt stress adaptation and dilution spot assay

The salt stress adaptation and dilution spot assay were conducted as described previously (29). Stationary-phase bacteria were diluted 1:100 in 5 mL BHI until the OD_600_ reached 0.5. Secondary inoculation (1:50) was carried out into BHI supplemented with 4.5% NaCl. Growth was monitored for 8 h. At the end of the time point (8 h), the cultures were frozen at −80°C for RNA isolation.

### Quantification of intracellular c-di-AMP concentration

Sterne strain and deletion mutants were inoculated in 5 mL LB broth and incubated for 16 h at 37°C. Secondary inoculation (0.1%) was performed in 5 mL BHI (Oxoid) (0.8% NaHCO_3_), and the cultures were incubated at 37°C for 18 h. *E. coli* strains were used to inoculate 4 mL M9 medium (35 mM KCl) containing 0.2% (wt/vol) glycerol and 0.02% (wt/vol) glucose (20, 27). The cultures were incubated overnight at 37°C and used to re-inoculate the same medium (without glucose) to an OD_600_ = 0.1. Then, the cells were washed and re-suspended in M9 medium (10 mM KCl) supplemented with 0.2% (wt/vol) glycerol, 0.005% arabinose, and 80 µM IPTG. The cultures were incubated for 18 h. Five milliliters of the cultures was harvested immediately by centrifugation at 4°C, and the cell pellets were extracted by the nucleotide extraction method reported previously (54). One microliter was used for high-performance liquid chromatography-tandem mass spectrometry (HPLC-MS/MS) analysis, which was performed on Agilent 1260 coupled to 6240A LC/MS/MS with an RP C18 column (150 mm by 2.1 mm, Waters XSelect HSS T3). The following buffers were used in the gradient program: buffer A: H_2_O; B: acetonitrile. The following protocol was used for separation: 0.01 min, 5% B; 3 min, 8% B; 6 min, 90% B; 9.1 min, 5% B; and 13 min, 0% B at a flow rate of 0.3 mL min^−1^. The intracellular c-di-AMP concentration was determined based on c-di-AMP (Sigma) standard plotting peak areas versus concentrations as nanogram of c-di-AMP per milligram dry weight *Bacillus anthracis.* Measurements were repeated in triplicates, with the same retention time as for c-di-AMP.

### Quantification of intracellular potassium concentration

Sterne strain and deletion mutants were inoculated in 5 mL LB broth and incubated for 16 h at 37°C. Secondary inoculation (0.1%) was performed in 5 mL BHI (Oxoid) (0.8% NaHCO_3_), and the cultures were incubated at 37°C for 18 h. Twenty-five-milliliter cultures were harvested, with the intracellular K^+^ concentrations determined using an atomic absorption spectrometer (204 Duo, Agilent, USA) as described previously (20). The intracellular concentrations of K^+^ were calculated using the following equation:


$${\left[K\right]}_{i}={\left[K\right]}_{t}÷W_{d}$$

in which [K]_i_ is the intracellular K^+^ in mg mg^−1^, [K]_t_ is the total K^+^ in mg, and W_d_ is the dry weight of the pellet in mg.

### Statistical analysis

Data in Fig. 2, 5C, and 6A and C were analyzed by two-tailed Student’s *t*-test. Data in Fig. 3 to 5A, 5B, and 6B and D were analyzed by using one-way analysis of variance followed by Tukey’s post-test analysis. Figure 7 was created with Biorender.com.
